# An explainable deep learning-based feature fusion model for acute lymphoblastic leukemia diagnosis and severity assessment

**DOI:** 10.3389/fmed.2025.1694024

**Published:** 2025-12-16

**Authors:** Hajra Khan, Muhammad Zaheer Sajid, Nauman Ali Khan, Muhammad Fareed Hamid, Ayman Youssef, Areesha Rehman, Nour Aburaed

**Affiliations:** 1 Department of Computer Software Engineering, Military College of Signals, National University of Science and Technology, Islamabad, Pakistan; 2 Department of Electrical Engineering and Computer Science, University of Missouri, Columbia, MO, United States; 3 Department of Computers and Systems, Electronics Research Institute, Cairo, Egypt; 4 Department of Pharmacy, Bahauddin Zakariya University Multan, Multan, Pakistan; 5 University of Dubai, College of Engineering and IT, Dubai, United Arab Emirates

**Keywords:** acute lymphoblastic leukemia (ALL), feature fusion, deep learning, Convolutional Neural Networks (CNN), image classification, image processing

## Abstract

Acute lymphoblastic leukemia (ALL) is a malignant blood disorder that primarily affects white blood cells, particularly in children, where early and accurate diagnosis is critical for effective treatment and recovery. This study introduces a novel deep learning framework, termed XIncept-ALL, for automated detection and classification of ALL severity levels. The model integrates pre-trained InceptionV3 and Xception networks through feature fusion blocks, enabling robust representation learning. To enhance performance, data auto-augmentation techniques were applied to address class imbalance and reduce overfitting. Furthermore, Grad-CAM visualizations were employed to highlight discriminative regions of ALL cell images, providing interpretability of the model's predictions and validating that the network focuses on clinically relevant features. The proposed system was evaluated using a newly developed private dataset, Pak-ALL, collected from Pakistani hospitals, along with additional datasets from reliable web sources. Extracted features were classified using an XGBoost classifier into four categories: Benign, Early, Pre, and Pro. Extensive experiments demonstrated the superior performance of the proposed framework, achieving an average accuracy of 99.5% on a challenging external dataset (Iranian dataset). The results highlight the efficiency, scalability, and practicality of the XIncept-ALL model for medical image analysis. By offering both improved classification accuracy and interpretable decision support via Grad-CAM, the proposed approach represents a significant advancement toward reliable computer-aided diagnostic systems for ALL, with strong potential to support clinical decision-making and improve patient outcomes.

## 1 Introduction

Acute lymphoblastic leukemia (ALL) is among the worst forms of malignancy and the most recognized type of leukemia. ALL is a blood cell cancer that begins in the bone marrow. It is characterized by an uncontrolled, accelerated growth of immature blood cells that affects both children and adults. The three primary components of blood have varying weights: red blood cells, plasma, and white blood cells (WBC). One WBC exists for every 100 red blood cells, or approximately 1% of the blood, which are WBC (1). WBC consists of five different types, and there is a standard count of each type from the total WBC count. Any change in the percentage of each type is considered an abnormality that can lead to immune disorders. These include basophils, eosinophils, neutrophils, lymphocytes, and monocytes disorders. According to the total WBC count, the standard counts for these cells are 60%, 30%, 5%, 4%, and less than 1%, correspondingly (2). Any abnormalities in these counts are considered a sign of cancer. The abnormality in the WBC count can be related to the production of other unwanted cells in this count. The overproduction of these cells, sometimes referred to as blasts or leukemic cells, causes them to hinder normal hematopoiesis and push out healthy leukocytes in the bone marrow. This makes it hard for the body to fight infections and regulate bleeding (3). In ALL, the malignant WBC are produced. These malignant cells attack normal blood cells and cause death. Malignant WBC also causes other types of deadly illnesses by traveling through the circulation, harming the liver, spleen, kidneys, brain, and other organs. Leukemia is a deadly disease and the most common disease among children and adults. Leukemia is a blood disorder linked to cancer that primarily arises from the bone's ability to produce immature white blood cells, which subsequently compromise the immune system (4). There are four categories of leukemia: acute lymphoblastic leukemia (ALL), chronic lymphocytic leukemia (CLL), acute myelogenous leukemia (AML), and chronic myelogenous leukemia (CML). ALL is one of the most prevalent cancers that affects kids and has a decent chance of being cured if discovered at an early stage. However, adults may also experience this, and the prospects of a recovery are minimal if found later on. Direct blood infusions into veins, chemotherapy, and any type of transplant that involves moving organs or tissues inside the body or from one person to another are all forms of treatment for acute lymphocytic leukemia. For doctors to detect this kind of disease, stained blood smear microscopy pictures are manually evaluated to provide the first diagnosis of ALL. This method can cause an error when diagnosing the disease and requires an expert eye to detect the disease. Moreover, this method is time-consuming. Computer-aided design tools are usually proposed to solve this issue. Automated analysis is provided by computer vision techniques, which can aid in the diagnosis of this illness by professionals. These tools help doctors diagnose the disease at an early stage and hence increase the patient's survival rate. Many tools in the literature provide an automated system for the diagnosis of this disease. In recent research studies, deep learning models have been used as the main component of such systems.

### 1.1 Information about acute lymphoblastic leukemia

The malignancy known as acute lymphoblastic leukemia (ALL) affects the uncontrolled proliferation of abnormal, immature lymphocytes and their progenitor cells. This finally leads to the substitution of additional lymphoid organs and parts of the bone marrow, resulting in a distinctive pattern of illness.

**Figure 1:**
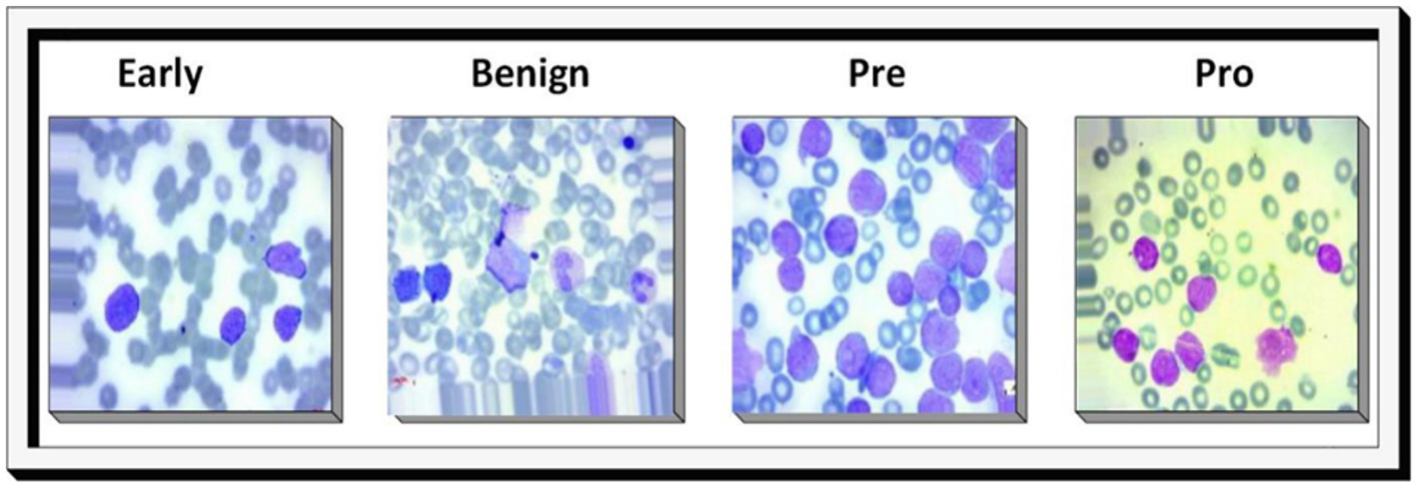
Sample images illustrating benign, early lymphoblastic leukemia, pre-acute lymphoblastic leukemia, and pro-acute lymphoblastic leukemia cases.

The following are some key medical facts about acute lymphoblastic leukemia (ALL):

- Signs and symptoms: bone marrow infiltration and/or extramedullary illness are frequently observed in patients with ALL. Anemia, thrombocytopenia, and neutropenia are symptoms of bone marrow failure that individuals experience when leukemic blasts replace their bone marrow. Additional people may exhibit fever, bleeding, blood clots, Disseminated Intravascular Coagulation (DIC), and anemia symptoms, such as tiredness, pallor, palpitations, heart murmur, and dyspnea, even with little exercise.
- Treatment: the subtype of ALL, the patient's age, and the existence of certain genetic abnormalities are some of the variables that affect how an individual with ALL is treated. Utilizing risk-adapted treatment methods has reduced medication toxicity while increasing cure rates. Treatment options for pediatric ALL include chemotherapy, stem cell transplantation, and radiation treatment.
- Prognosis: the patient's age, the kind of ALL, and the existence of specific genetic abnormalities are among the variables that impact the prognosis of ALL. Due to improvements in diagnosis and treatment, children with acute lymphoblastic leukemia now have a 90% overall cure rate (5). Figure 1 shows different stages of ALL.

Understanding the medical information behind ALL is crucial for healthcare professionals and patients to develop effective treatment plans and manage the physical and emotional challenges associated with this disease.

### 1.2 Research motivation

White blood cell classification has been the focus of extensive research, with numerous approaches reported in the literature (Table 1). Earlier methods primarily relied on a combination of traditional machine learning models and image processing techniques. For instance, in Jagadev and Virani (6), a conventional strategy using handcrafted feature extraction combined with a Support Vector Machine (SVM) classifier was proposed. Similarly, the authors in Amin et al. (7) employed geometrical and statistical features of nuclei to train an SVM for detecting ALL. Another study (8) also explored handcrafted quantitative features for automatic cell image recognition. In Mahmood et al. (9), various models were tested for pediatric ALL detection, and the Classification and Regression Tree (CART) model was found to be the most suitable for the dataset under study. Similarly, Bodzas et al. (10) presented an automatic ALL classification method based on machine learning and digital image processing, which involved a three-phase filtration algorithm and extraction of 16 handcrafted features fed into SVM and Artificial Neural Network (ANN) classifiers. Although these methods achieved promising results, they are limited by their reliance on manually engineered features, which often fail to generalize across diverse datasets. Recent studies have demonstrated the effectiveness of deep learning-based approaches, particularly pretrained Convolutional Neural Networks (CNNs), in achieving superior performance on various cell classification tasks. These models can automatically learn hierarchical representations from raw images, eliminating the need for handcrafted feature design. Motivated by these advancements, this study proposes a novel deep learning model for ALL classification that leverages feature fusion from two pretrained CNN architectures. Furthermore, the model is evaluated using a newly collected dataset, offering a fresh benchmark for future studies.

**Table 1:** Contributions and outcomes of different articles for the classification of acute lymphoblastic leukemia (ALL) images.

**Authors**	**Publication year**	**Contribution**	**Results**
Vaghela HP (1)	2020	Using CNN for the classification of ALL disease.	99.5% on average across all trials.
Alexandrea Bodzas (28)	2020	Using a neural network and a support vector machine as a classifier for their own dataset.	The neural network model achieved 97.5% which is better than the SVM model.
De Oliveira (29)	2021	Small modifications to different neural network models (VGG16, VGG19, Xception). Image augmentation methods were used to balance datasets.	Accuracy is not reported; however, the model achieves an F1-score of 92.6.
Larissa Ferreira Rodrigues (30)	2022	Using a genetic algorithm to optimize a deep residual neural network.	98.46%
Ghada Atteia (38)	2022	Tuning the hyperparameters of CNN using the Bayesian optimization algorithm.	100% accuracy on ALL-IDB dataset.
Ahmad Almadhor (27)	2022	Testing ensemble voting and machine learning techniques like RF, SVM, KNN, and NB classifiers using predefined CNN architectures (VGG16, ResNet50, or ResNet101) for feature extraction methods.	Superior performance of the SVM model over other techniques, reaching 90% on C-NMC 2019, is reported.
Ibrahim Abdulrab Ahmed (33)	2023	After merging DenseNet121-ResNet50-MobileNet's deep features map, RF or XGBoost was used to classify the data.	Accuracy 98.8% on C-NMC 2019 Accuracy 100% on ALL-IDB2
Tulasi Gayatri Devi (24)	2023	Propose a novel GBHSV-Leuk to detect and classify ALL disease. The method consists of two stages Gaussian blurring technique and the HSV technique.	Using the ALL-IDB1 public dataset, the accuracy was 95.41%.
Jose Luis Diaz Resendiz (31)	2023	An explainable model to solve the problem of black-box. Using a novel image segmentation technique combining image processing and U-net techniques.	Accuracy 98.51% on ALL-IB2.
M.Anline Rejula, (34)	2023	Classification of ALL using improved ANFIS	Accuracy 97.4% on the ALL image dataset.
Sajida Perveen (38)	2024	Weighted ensemble learning technique using a Residual Convolutional Neural Network (ResNet-152) as the base learner to identify ALL from hematogone cases and then determine ALL subtypes.	The proposed platform achieved comparatively high accuracy (99.95%).
Lida Zare (35)	2024	The use of six graph convolutional layers and one softmax layer.	The model's accuracy remained above 90%, even at a Signal-to-Noise Ratio (SNR) of 0 dB.

### 1.3 Paper contributions

In this research, a novel system (XIncep-ALL) is designed to address the challenging task of ALL classification. It does this by classifying data into Benign, Early, Pre-Acute, and Pro-Acute using the proposed architecture. This task is considered challenging because ALL can be present with a wide variety of cell types and shapes. Furthermore, there is a small apparent difference between normal and ALL cells.

The following points are the XIncep-ALL system's important contribution points:

- A novel dataset of 9,395 ALL images was collected from Pakistani hospitals and online sources. This large dataset enables high classification accuracy and is available for other researchers to use.
- A multilayer design combining Xception and Inception blocks through feature fusion was developed, achieving higher accuracy than existing methods.
- Deep learning with feature fusion was applied to capture discriminative features, resulting in superior performance compared to prior work.
- Multiple experiments confirm that XIncep-ALL consistently outperforms state-of-the-art models, largely due to training on the comprehensive Pak-ALL dataset.

### 1.4 Paper organization

The remaining sections of this study are organized as follows: Section 2 explores the review of literature related to the study topic. Section 3 presents the methodology and techniques. Section 4 discusses the experimental findings and compares our results with cutting-edge research results. Section 5 introduces the state-of-the-art comparison. Section 6 gives the manuscript's conclusion, while Section 7 discusses the main challenges in this research area, and Section 8 discusses the Challenges and Future Works.

## 2 Related work

This section summarizes papers from the literature that apply deep learning models to the ALL classification problem. In Macawile et al (11), the authors use pretrained Deep learning models (GoogleNet, AlexNet, and ResNet-101) to build a system for WBC classification. The authors compare the models in terms of classification accuracy. In Hegde et al. (12), the authors propose a comparison of deep learning architectures with traditional methods for digital image processing with regard to the classification of WBC. The results prove the superior performance of deep learning architectures over traditional methods. A customized CNN architecture for classifying WBC cells is presented in Sharma (13). High accuracy scores are obtained by this design in multiclass and binary classification scenarios. In Habibzadeh et al. (14), a novel system that combines ResNet and Inception networks for WBC classification is proposed. The proposed work in this study is divided into two stages. The First stage is the pre-processing stage, which involves three pre-processing steps (color distortion, bounding box distortion (crop), and image flipping and mirroring). The second stage of this study is using the two deep learning models as hierarchical topological feature extractors. A model that uses the most important features of a color picture to detect ALL is provided in Muntasa and Yusuf (15). The suggested model consists of four primary stages: accuracy measurement, feature extraction, segmentation, and improvement. The ALL-IDB dataset's highest accuracy was achieved using the suggested approach. The authors of Shafique and Tehsin (16) suggest a comparison study of several approaches for the early identification of ALL. This study compares and analyzes the various phases of the diagnostic process. A methodology for recognizing ALL using WBC microscopic pictures is presented in Bhuiyan et al. (17). For classification, a total of four distinct ML models are employed, and the results are compared. The authors find that the SVM model provided the highest accuracy for the used dataset based on the experimental results. The work in Acharya and Kumar (18) provides a unique method for segmenting the WBC's cytoplasm and nucleus. The proposed study evaluates several approaches utilized in the literature to segment WBC. The resulting microscopic images are then classified into the four classes of ALL using supervised classification models that are constructed to extract characteristics. The authors of Kasani et al. (19) suggest using an aggregated pretrained CNN algorithm to identify ALL in microscopic WBC images. The authors prevent overfitting by employing a number of data augmentation strategies. Together, the VGG19 and NASNetLarge make up the network, which is utilized for classification. The total accuracy generated by the final ensemble was higher than that of any of the individual networks. In Sahlol et al. (20), Ratley et al. (21), and Salah et al. (22), a thorough overview of the methods and trends used now to identify leukemia from microscopic images is provided.

In Anwar and Alam (23), a novel automated model for the diagnosis of acute ALL using a CNN is presented. The model works on labeled blood images obtained from a database called ALL-IDB. Through a five-fold validation process, the model was trained on 515 photos, achieving a 95.54% accuracy rate. After testing the model on the remaining 221 photographs, it achieved an accuracy of approximately 100% in most of the trials, with an average accuracy of 99.5%. The approach effectively uses unprocessed data without the requirement for pre-processing or segmentation. Thus, using this technique can help pathologists accurately diagnose ALL.

In Devi et al. (24), a novel system for ALL classification is proposed. The system's initial step is image pre-processing. The authors use the Gaussian Blurring (GB) method to enhance the image before classification. The picture is then subjected to a segmentation phase employing the Hue Saturation Value (HSV) technique and a morphological stage. Applying these steps, the system achieved an accuracy of 96.30% on the author's collected dataset while achieving 95.41% accuracy on the ALL-IDB1 dataset.

In Zakir Ullah et al. (25), a novel diagnostic model based on CNN is proposed. The suggested approach is built on deep learning processing of medical images. This study presents a CNN-based non-invasive diagnosis technique. The proposed approach, which consists of a CNN-based model, uses a visual geometry group from Oxford (VGG16) model combined with an attention module called Efficient Channel Attention to extract better quality deep features from the image dataset, improving feature representation and classification outcomes. The proposed technique proves how the ECA module helps in minimizing morphological similarities between photos of healthy and cancerous cells. Using a range of augmentation techniques also increases the quantity and quality of training data.

In Sampathila et al. (26), a novel algorithm for the classification of leukemic cells from healthy and non-healthy blood cells is proposed. The algorithm is implemented with a CNN to predict the disease from microscopic images. The trained system reached a maximum accuracy of 95.54%. The system can be used for the detection of ALL in blood.

In Almadhor et al. (27), an ensemble automated prediction model is proposed. The model consists of four different machine learning algorithms: Naïve Bayes (NB), K-Nearest Neighbor (KNN), SVM, and Random Forest (RF). The system is built first using a data pre-processing step. The image is cropped. Then, feature extraction is performed using pre-trained deep learning models (VGG19, ResNet50, or ResNet101). The MinMaxScaler normalization technique is used to scale the data. As feature selection methods, the authors employed random forest, recursive feature removal, and Analysis of Variance (ANOVA). Ensemble voting of the classifications of machine learning algorithms is applied. Results show that SVM can reach 90% accuracy.

In order to automatically detect ALL cells, a novel method based on ML algorithms and digital image processing algorithms is presented in Bodzas et al. (28). To get the best segmentation results, a pre-processing step using three-phase filtering algorithms is suggested, followed by a segmentation step to tackle this issue. Two machine learning classifiers (ANN and SVM) are proposed for the classification of images. The system achieved 98.25 and 100% accuracy, respectively. In de Oliveira and Dantas (29), novel modifications to conventional neural network architectures are proposed. These modifications result in a good performance in the classification of malignant leukocytes. Some of the image processing steps applied to images are inserting salt-and-pepper noise, spinning, fading, and shearing. The models used for testing are VGG16, VGG19, and Xception. The proposed model achieves an F1-score of .926 using data augmentation.

In Rodrigues et al. (30), the authors propose a new hybrid model for the classification of ALL diseases. In this model, the residual CNN, ResNet-50V2, is trained with the GA method to determine the optimal hyperparameters that maximize accuracy. This method of using optimization algorithms is usually used instead of tuning these values manually, which is a time-consuming task and does not always result in the best accuracy.

In Diaz Resendiz et al. (31), an explainable system for ALL classification is proposed. This study proposes a novel deep learning model for ALL classification that is extremely accurate and understandable due to the visual explainability added to the model. This study suggests a strong WBC nuclei segmentation and uses the Explainable AI technique to overcome the black box problem. The segmentation of WBC is made possible through the integration of image processing techniques and U-Net techniques, which enhances system performance.

This study (32) proposes a variant of deep neural networks called the ALNett model to categorize images of tiny white blood cells. The proposed model consists of different layers (Convolution, max-pooling, and normalization) that are used to accurately predict ALL by taking powerful features extracted from the microscopic blood images. These layers also give richer structural and contextual data. The model's performance was compared with other models, such as VGG16, ResNet-50, GoogleNet, and AlexNet, based on metrics such as precision, recall, accuracy, F1 score, loss accuracy, and Receiver Operating Characteristic (ROC) curves. Experimental results showed that the proposed ALNett model achieved a maximum accuracy in classification of 91.13% and an F1 score of 0.96 with less computing complexity. ALNett outperformed the other training networks and showed promising ALL classification.

In Ahmed et al. (33), a new deep learning model system is proposed to classify the images of C-NMC 2019 and ALL-IDB2 datasets with high efficiency and accuracy. In the proposed system, blood micrographs were enhanced, and the WBC-only areas were then extracted using the active contour approach and sent to three different CNN models (ResNet50, DenseNet121, and MobileNet) for additional analysis. In this study, the hybrid model was constructed using CNN-RF and CNN-XGBoost, which is the first work for analyzing ALL images from two different datasets. Deep feature maps are extracted in this hybrid model using the DenseNet121, ResNet50, and MobileNet models. With redundant and insignificant features, these CNN models generate large feature counts. In order to pick highly representative features, CNN deep feature maps were input into the Principal Component Analysis (PCA) technique. From there, the features were given to the classifiers (RF and XGBoost) for classification. In Rejula et al. (34), Adaptive Network-Based Fuzzy Inference Systems (ANFIS) are used to handle this classification of leukemia cases. ANFIS is a useful tool because of its reputation for function approximation. Nevertheless, its accuracy could be improved. In this study, an enhanced ANFIS (I ANFIS) model is suggested in order to get over this restriction. The model in this study compares training and test feature data and then makes use of Euclidean distance to predict leukemia data.

## 3 Methodology

**Figure 2:**
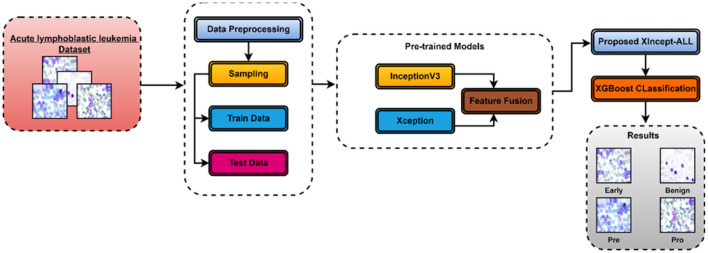
Block diagram of a proposed XIncept-ALL model.

This section explains the XIncept-ALL proposed model to classify and detect ALL diseases according to their severity. Figure 2 shows the suggested architecture of this study. To improve picture visualization, a pre-processing procedure is applied once the image is first obtained using appropriate image processing techniques. Subsequently, the designed deep learning model is deployed to extract features from images and identify the most relevant features for acute lymphoblastic leukemia (ALL) classification. Finally, the picture is classified into several ALL-related categories using an XGBoost classifier. The model introduced in this study is called the XIncept-ALL model by the authors. The XIncept-ALL system leverages the InceptionV3 model and the Xception model. Deep learning is crucial in extracting meaningful features for the Computer-Aided Diagnosis (CAD) systems. Transformation learning is employed to retrain a previously learned model on an ALL dataset. The XIncept-ALL architecture, designed for extracting relevant ALL features from images, comprises six steps as depicted in Figure 2. The features extracted from both the InceptionV3 model and Xception model are combined through a feature fusion block. The classifier layer is incorporated as the final step to identify the image as Benign, Early Lymphoblastic Leukemia, Pre-Acute, and Pro-Acute. The XGBoost classification process thereby enhances overall classification outcomes. In addition, to ensure interpretability and clinical reliability, Gradient-weighted Class Activation Mapping (Grad-CAM) was employed. Grad-CAM highlights the most discriminative image regions that influence the model's decision, enabling visualization of critical features such as nuclei and morphological abnormalities. By generating heatmaps normalized between 0 and 1 and overlaying them onto the original images, Grad-CAM provides meaningful insights into the decision-making process of the XIncept-ALL system.

The proposed model is designed with a focus on enhancing the classification accuracy and robustness by integrating multiple CNNs as feature extractors. The proposed model consists of three DL modules: the cooperative CNN module, the feature fusion module, and the feature classification module. Each module plays a crucial role in the overall functionality of the model, contributing to its ability to extract and combine features effectively. The following sections describe these modules in detail, along with the mathematical formulations that govern their operations.

The first stage of the model involves the extraction of significant features from input images using two pre-trained CNN models: Xception and InceptionV3. These models are well-established in the field of computer vision and are known for their ability to capture complex patterns in image data. By leveraging these models, the proposed approach avoids the computational burden associated with training new CNN models from scratch.

Let the input image be denoted as:


$$\begin{array}{l} X\varepsilon R^{\left(h\times w\times c\right)} \end{array}$$ (1)

where h represents the height, w represents the width, and c represents the number of channels (e.g., three for RGB images). The Xception and InceptionV3 models, which are pre-trained on the ImageNet dataset, are fine-tuned using Transfer Learning (TL) to extract features that are pertinent to the specific task at hand.

The Xception model processes the input image X to produce a feature vector:


$$F_{Xception\;}=\;R^{d_{1}}$$ (2)

This can be mathematically expressed as:


$$F_{Xception\;}=\;F_{Xception}\;(X;\theta_{Xcpetion})$$ (3)

where **F**_**Xception**_ is the function representing the Xception model's feature extraction process, and **Θ**_**Xcpetion**_ denotes the model parameters (weights and biases).

Similarly, the InceptionV3 model extracts a feature vector $F_{InceptionV3}\;\in \;R^{d_{2}}$from the input image X. This extraction is represented by the following equation:


$$F_{InceptionV3}=F_{InceptionV3}(X;\theta_{InceptionV3})$$ (4)

where **F**_**InceptionV3**_ is the function for the InceptionV3 model, and _**InceptionV3**_ represents its parameters. These feature vectors **F**_**Xception**_ and **F**_**InceptionV3**_ capture various aspects of the input images, with each model focusing on different patterns and details due to their unique architecture.

Once the features have been extracted by the Xception and InceptionV3 models, the next step involves combining these features into a single, unified representation. This process is known as feature fusion and is critical for enhancing the overall performance of the model. By fusing the features, the model can leverage the strengths of both CNNs, thereby creating a more comprehensive feature representation.

The feature vectors **F**_**Xception**_and **F**_**InceptionV3**_ are concatenated to form a unified feature vector **F**_**fusion**_. The concatenation operation is denoted as:


$$F_{fusion}=\text{Concat}(F_{Xception\;},\;F_{InceptionV3})$$ (5)

where $F_{fusion}\varepsilon \;R^{\left(d_{1}+d_{2}\right)}$ is the combined feature vector that serves as the input for the subsequent classification stage. This fusion process allows the model to integrate the diverse features extracted by the Xception and InceptionV3 models, resulting in a richer and more varied feature representation.

The fusion of features from multiple models offers several advantages. First, it captures a broader spectrum of patterns within the data, as different CNNs are typically sensitive to different types of features. Second, by combining the strengths of multiple models, the fused feature vector can provide a more robust representation, mitigating the risk of overfitting and improving the model's generalization capacity.

The final stage of the proposed model is the feature classification module, where the fused feature vector **F**_**fusion**_is passed through a series of fully connected (dense) layers to produce the final classification output. The classification process assigns probabilities to each class and distinguishes between the four distinct classes: Benign, Early Lymphoblastic Leukemia, Pro-Acute, and Pro-Acute.

The fused feature vector **F**_**fusion**_ is first processed by a dense layer, which applies a linear transformation followed by a non-linear activation function. The output of the first dense layer *H*_1_ is given by:


$$\begin{array}{l} H_{1}=\sigma \left(W_{\text{1}}\;F_{\text{fusion}}+b_{\text{1}}\right) \end{array}$$ (6)

where $W_{1}{\varepsilon R}^{n_{1}(d_{1}+d_{2})}$ is the weight matrix, **b**_**1**_ ε ${\text{R}}^{{\text{n}}_{1}}$ is the bias vector, and σ is the activation function, typically Rectified Linear Unit (ReLU). This layer introduces non-linearity into the model, allowing it to capture complex relationships between the features. The output **H**_**1**_ is then passed through an additional dense layer, which further refines the feature representation. The output of this second dense layer **H**_**2**_ is given by:


$$\begin{array}{l} H_{2}=\sigma \left(W_{2}\;H_{1}+b_{2}\right) \end{array}$$ (7)

where $W_{2}{\varepsilon R}^{\left(n_{1}*n_{2}\right)}$ is the weight matrix, and **b**_**2**_ ε **R**_**2**_ is the bias vector.

The final classification is performed using the XGBoost classifier.

### 3.1 Dataset

**Figure 3:**
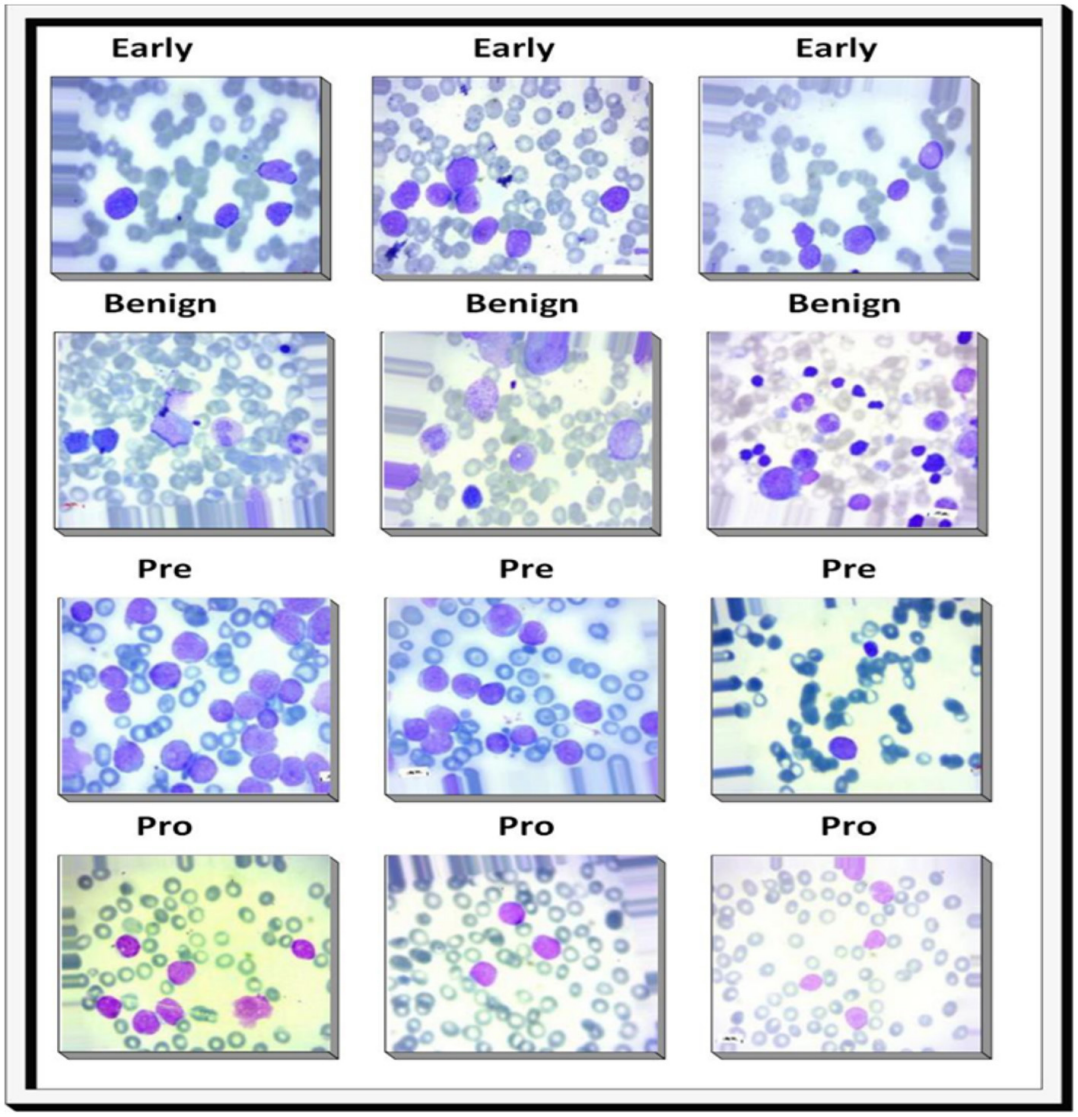
Sample images from the dataset: the first row illustrates the early lymphoblastic leukemia stage, the second row shows the Benign stage, the third row depicts the pre-acute stage, and the fourth row represents the pro-acute stage.

The dataset was collected from well-known online sources and Pakistani Hospitals. Comprising 9,395 PBS images derived from 500 subjects suspected of ALL, these images were prepared and stained by adept laboratory professionals. The dataset has four classes: Benign, Early, Pre, and Pro. In this study, we have four categories for training and testing the proposed model. The categories are Benign, Early, Pre, and Pro. The Benign class represents normal or non-malignant lymphoid cells. The Early stage corresponds to initial leukemic transformation. The Pre stage reflects more advanced leukemic progress, with higher blast counts representing an intermediate level of severity. Finally, the Pro stage corresponds to progressive or aggressive disease. All images were captured with a Zeiss camera under a microscope at 100 × magnification and saved in JPG format. Definitive cell type and subtype determinations were made by a specialist employing the flow cytometry tool. Additionally, to enhance analysis, segmented images were generated through color thresholding-based segmentation in the HSV color space, providing a valuable resource for further investigation and interpretation. The dataset is shown in Figure 3. The data split used in this investigation for ALL is described in Table 2. Table 3 shows the dataset's main components.

**Table 2:** Data distribution of ALL datasets across benign, early, pre-acute, and pro-acute classes.

**Stages**	**Number of images**	**Size of images**
Benign	2,452	224 × 224
Early	2,785	224 × 224
Pre	2,262	224 × 224
Pro	1,896	224 × 224
Total	9,395	–

**Table 3:** Dataset division table.

**Dataset name**	**Source**	**Number of images**
Iranian dataset	Taleqani Hospital's bone marrow laboratory in Tehran	3,256 Peripheral Blood Smear (PBS) images obtained from 89 subjects suspected
Kaggle dataset	Kaggle	This dataset consists of 150 images, with 50 images in each class
Private	Pakistani hospitals	5,990
Combined dataset (PAK-ALL)	Merged Pak-ALL + public datasets	9,395

### 3.2 Pre-processing and augmentation

Pre-processing ALL pictures involves a complex set of steps meant to improve the raw data images. Initially, the raw data of the photos is cleaned. This cleaning included the handling of erroneous or missing pixel values and the removal of outliers within the data. The next step is zooming in on cells and cropping the investigated area. Next, data augmentation techniques are used to increase the data and solve the dataset imbalance problem. These augmentation steps included Rotation and horizontal flipping. The next step is correcting contrast and boosting using the CAMSR approach.

A detailed account of the various preparatory procedures, including data augmentation approaches, is presented in Table 4, providing pre-processing steps parameters.

**Table 4:** Pre-processing steps applied to the dataset images.

**Pre-processing step**	**Parameters used**
Zoom range	0.2
Crop	True
Width shift range	0.2
Rotation range	15
Vertical flip	False
Horizontal flip	True

**Figure 4:**
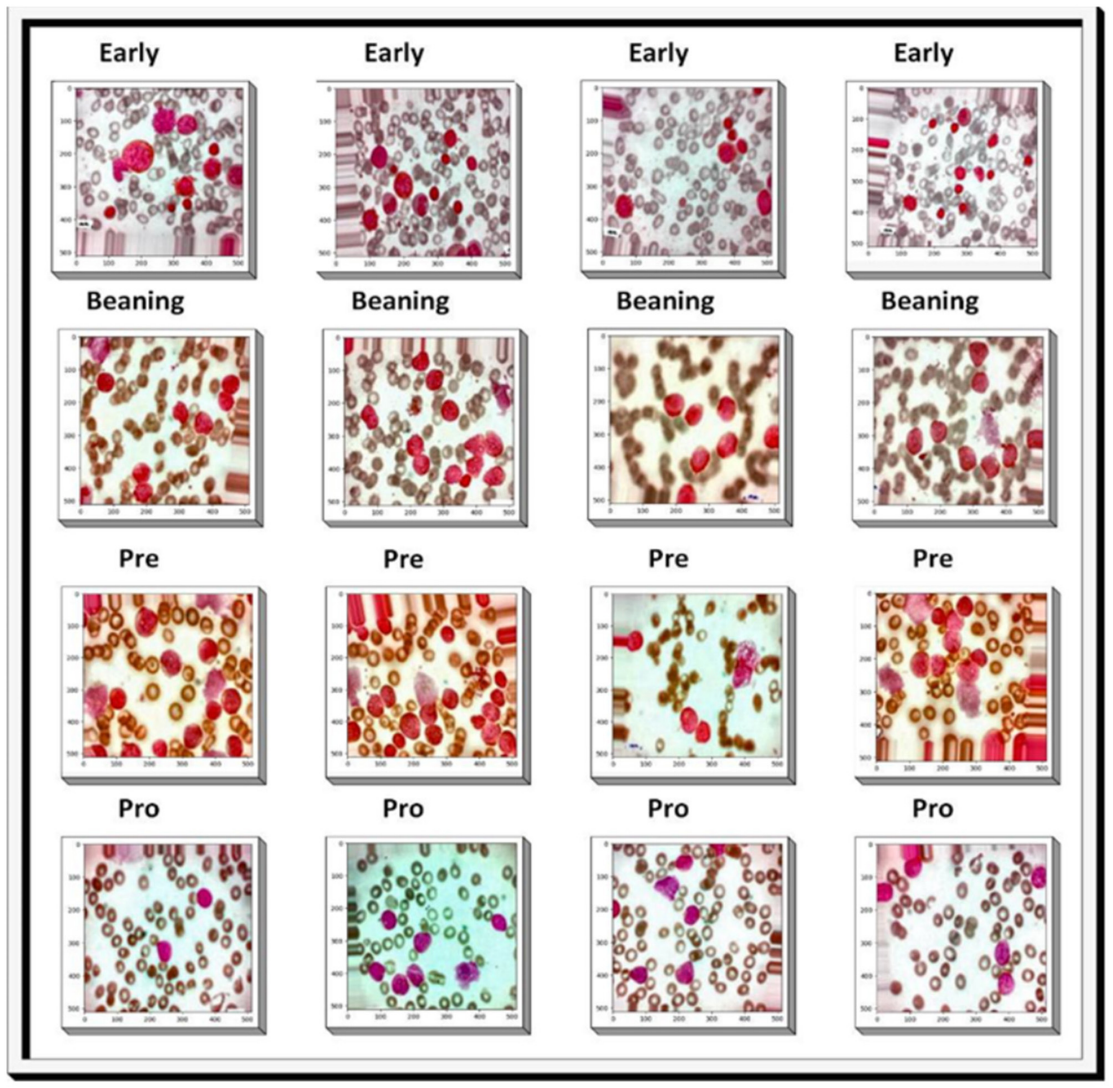
Results of image pre-processing steps to enhance contrast and adjust noise.

Figure 4 illustrates the results of the pre-processing steps applied to images before being inserted into the designed model. It was necessary to crop out undesirable portions of the image in order to preserve only the desired portions of the image. Contrast adjustment was performed to change the image brightness levels. Horizontal was used to reorient the image along the respective axes. The process of panning is performed on the image. Furthermore, to provide depth and texture to a picture, pixels were moved up or down using the embossing process. The combined impact of these procedures enhanced both image quality and classification accuracy.

### 3.3 Proposed architecture background

Deep Learning (DL) algorithms can handle huge volumes of data. This allows the DL algorithm to have excellent processing skills and effective classification of ALL images. These algorithms can be used to handle every stage of the classification problem effectively. DL algorithms can be used in data pre-processing, model design, hyperparameter tuning, and the selection of architectural parameters. In this study, two models (InceptionV3 and Xception) are proposed to build a model for ALL classification. These two models have several noteworthy benefits, such as their superior performance on the ImageNet dataset and enhanced feature extraction design. In addition, these models corrected residual connections and linear units, improved the quality of feature representation, and solved the gradient vanishing issue. To lower the chance of overfitting, they additionally employ global average pooling and dropout layers. Adding Batch Normalization layers also expedites the training process, approaching anomaly identification in ALL pictures more effectively and efficiently. We will provide a brief description of the sophisticated models used in this study in the paragraphs that follow.

#### 3.3.1 InceptionV3 architecture

**Figure 5:**
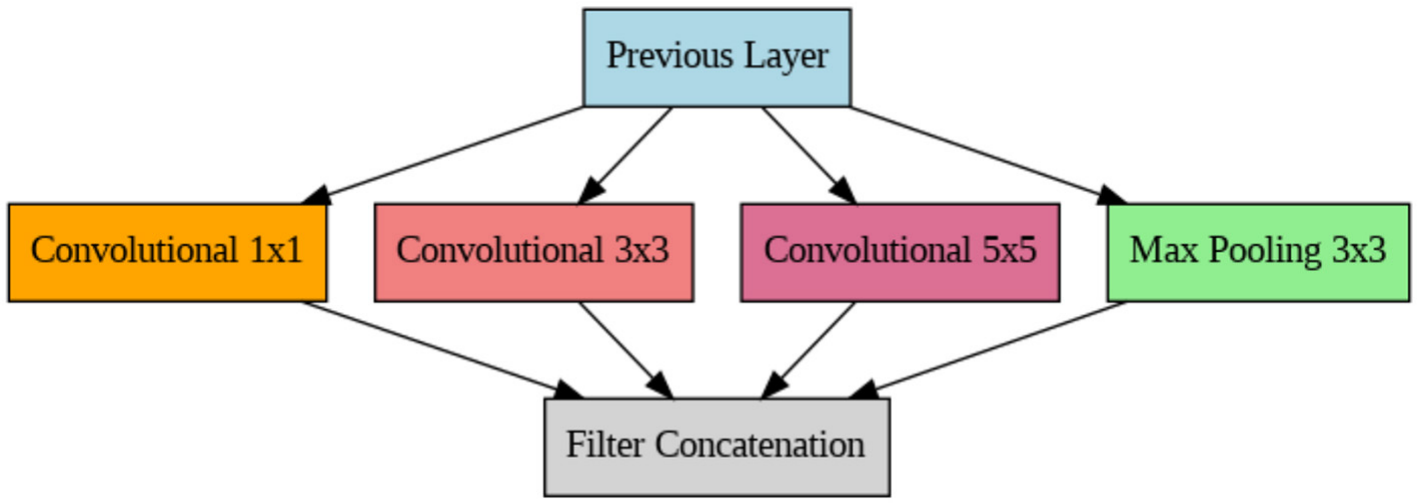
Block diagram of the InceptionV3 model.

A complex deep neural network design called InceptionV3 is capable of extracting characteristics at various scales (35). InceptionV3 incorporates factorized convolutions and parallel processing paths to model multi-scale visual information. This architecture achieves high efficiency by balancing depth, width, and computational complexity (40). The three primary structural components of this design are the final layers, initiation blocks, and stem. The inception block consists of convolutional layers and inception modules. Inception blocks focus on deepening the network. In this model, the convolutional layers deepen the network, and the inception modules extract features at different scales. The last layer, which minimizes the spatial dimension of the feature maps and generates the model's output, usually consists of many convolutional layers and a Global Average Pooling (GAP) layer. The final classification is produced by a completely linked layer, which receives the output from the previous layers. Figure 5 shows the block diagram of the InceptionV3 model.

#### 3.3.2 Xception architecture

**Figure 6:**
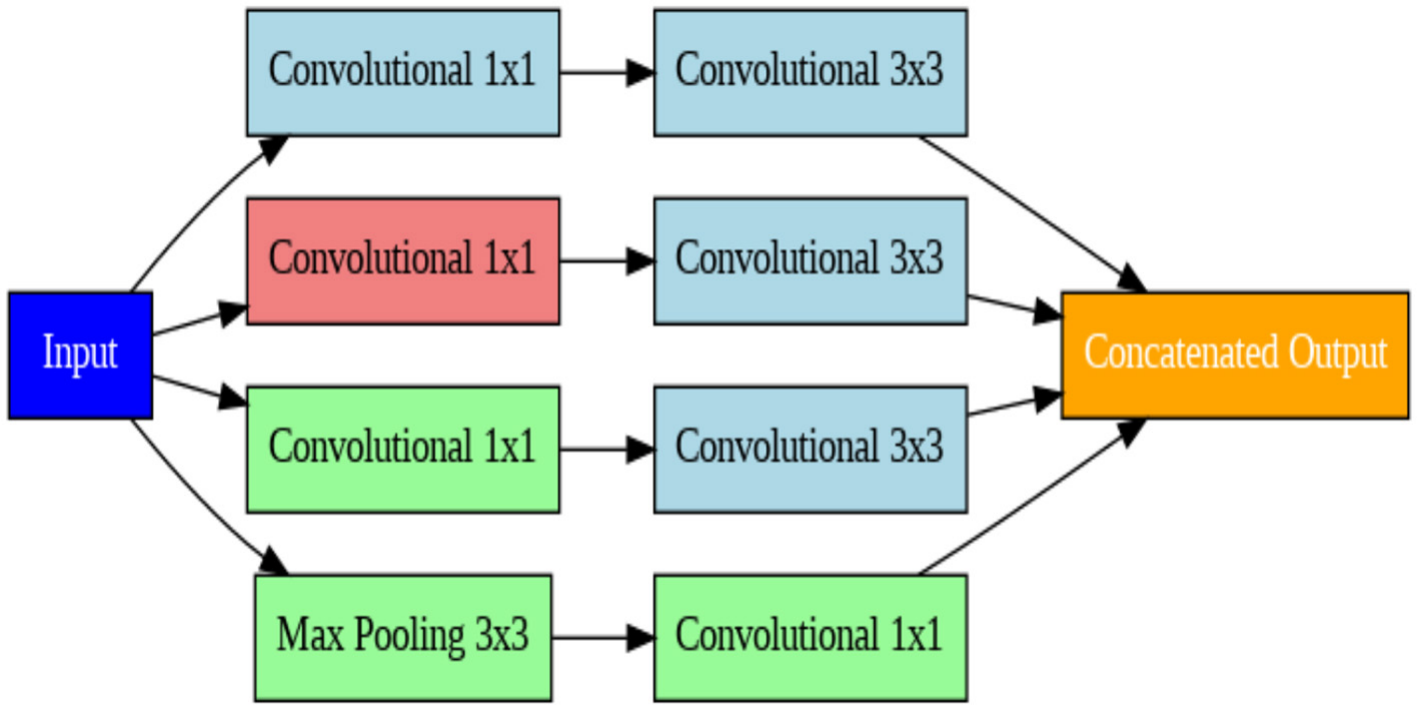
Block diagram of the Xception model.

The Xception model is another sophisticated deep CNN model (36). This model takes advantage of depth-wise separable convolution, a more efficient convolutional procedure. Depthwise separable convolutions can be interpreted as a limiting case of Inception modules with an arbitrarily large number of parallel towers. Leveraging this insight, the Xception architecture replaces Inception blocks with depthwise separable convolutions, achieving superior accuracy to Inception V3 with the same parameter budget (39). This divides the convolution process into two steps: a depthwise convolution that applies one filter to each input channel, and a pointwise convolution that combines the output of the depthwise convolution. This reduces the number of parameters in the model and speeds up processing. The architecture facilitates the spatial dimensions of the feature map and inhibits overfitting by incorporating completely connected layers, an optional dropout layer, and Global Average Pooling (GAP layer) (37). Figure 6 displays the block diagram of the Xception model.

### 3.4 The proposed fusion architecture

**Figure 7:**
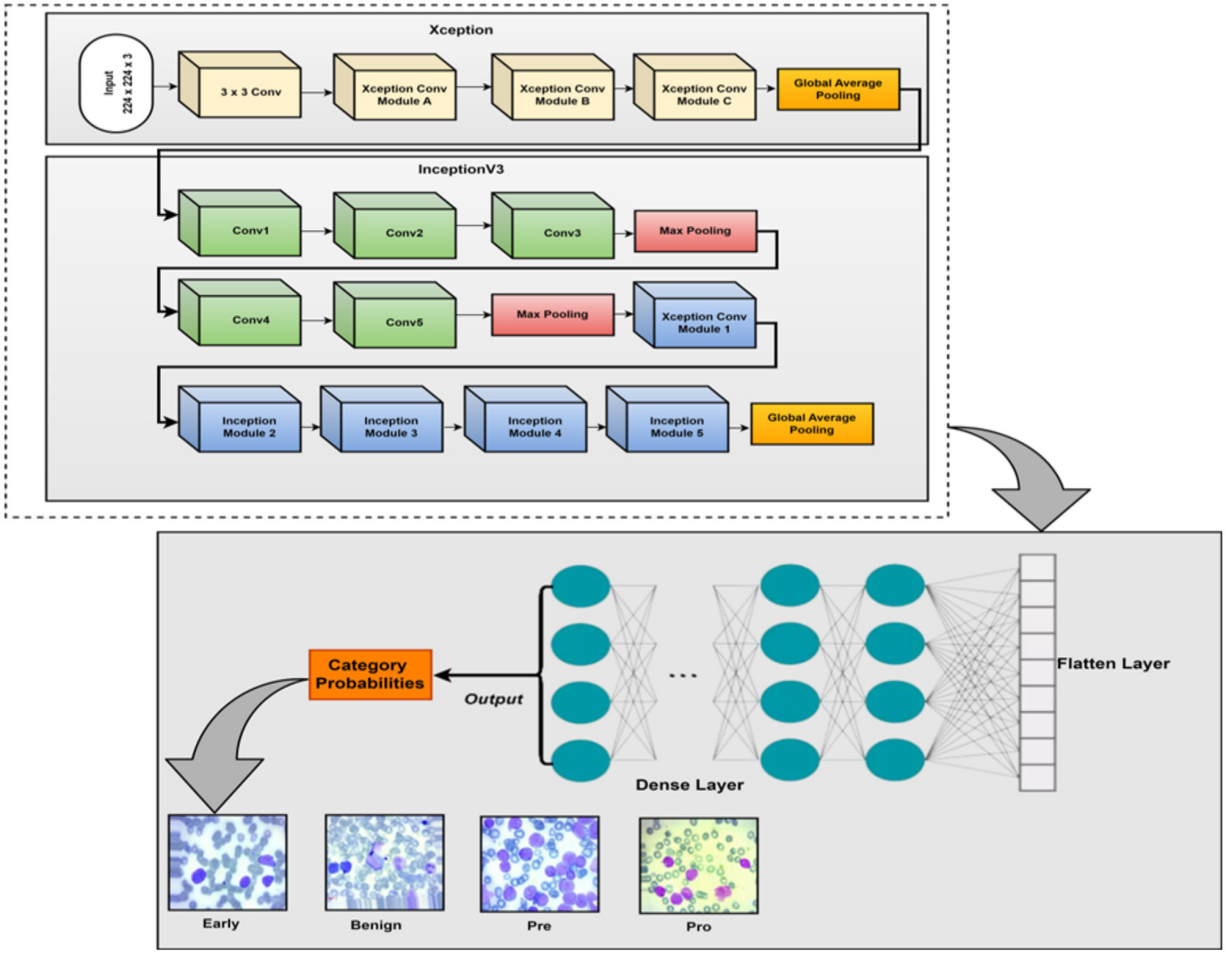
Proposed architecture block diagram.

The proposed model is structured with three modules, each contributing to its comprehensive functionality: a cooperative CNN module, a feature fusion module, and a feature classification module. The visual representation of the Fusion model is encapsulated in Figure 7. The model leverages the strengths of two pre-trained CNN models, Xception and InceptionV3, to precisely extract relevant features. The Xception and InceptionV3 models are initially trained on the ImageNet dataset. The models are fine-tuned through TL to be able to work on ALL images. Subsequently, the feature fusion module combines the extracted features from the cooperative CNN into a unified feature pool, serving as the input for the subsequent feature classification stage. A variety of machine learning classifiers can be used by the feature classification module. This module ascribes class probabilities and distinguishes between four distinct classes: Benign, Early Lymphoblastic Leukemia, Pro-Acute, and Pro-Acute. Crucially, the weights of the features are dynamically trained during this classification process. The Fusion model is a powerful and adaptable tool for behavior detection and classification since it has many beneficial features and capabilities.

The model provides an adaptable approach to combining different CNN models without training them together. The proposed DL models are trained independently on datasets of relevance. Afterward, feature fusion is used to smoothly integrate the features that were retrieved from these two models into the single feature pool.

This approach has the advantage of high resource efficiency and a small time needed for training. This is because each model is pre-trained independently, and there is no need to train the complex model; only fine-tune on the desired dataset. The proposed approach captures features from two different DL models. The two models combined produce an enhanced feature representation that surpasses that extracted by a single DL model. This is supported by the realization that different models have different degrees of accuracy in identifying particular features or patterns in the data. Combining these different features makes it easier to create a feature representation that is more comprehensive and varied, covering a wider range of patterns and relationships that are present in the data.

Moreover, the deliberate combination of several models accomplishes both the objectives of reducing the likelihood of overfitting and enhancing the model's ability to generalize. This integration improves the model's ability to extract patterns from unknown data, making it more resilient and adaptive. It also strengthens the model's robustness by reducing the effect of distinctive features that come with separate models.

In our investigation, we also explored the efficacy of the XGBoost algorithm for the task of recognizing features associated with ALL. The algorithm, outlined in detail as Algorithm 1, employs a gradient boosting framework with decision trees as base learners. XGBoost is particularly well-suited for binary classification tasks, and we configured it with hyperparameters such as the learning rate (η), regularization term (λ), and the number of trees (*T*).

Algorithm 1 Detailed steps of the XIncept-ALL model for feature extraction.

 ***Input***
 **Image dataset with labeled classes: {(*x*1, *y*1), (*x*2, *y*2), …, (*xN, yN*)}**
 ***Output***
 Trained a combined model for image classification
 *Step 1*
 Initialize Xception and InceptionV3 base models: *MXcep* ← = *Xcep (weights = “imagenet”, include_top = False, input_tensor = Input(shape = (224,224,3))) and MIcep* ← *Incep(weights = “imagenet”, include_top = False, input_tensor = Input(shape*= (224,224,3))).
 *Step 2*
 *FXcep ← Xcep_last_layer(Xtrain)* Get features from the desired layer in Xception.
 *Step 3*
 *FIncep ← Incep_last_layer(Xtrain)* Get features from the desired layer in InceptionV3.
 *Step 4*
 *DXcep ← Decep(FXcep, units = 64, activation = “relu”)* Dense layer for Xception features.
 *Step 5*
 •*OXcep ← Dense(DXcep, units = 2, *kernel*_*regularizer*_ l2(0.01), activation =* linear) Linear output layer for Xception features. •*OIncep ← Dense (DIncep, units = 2, kernel_regularizer = l2 (0.01), activation = “linear”)* Linear output layer for InceptionV3 features.
 *Step 6*
 •*MXcep ← Model(inputs = *Xcep*_*inputs*_, Outputs = OXcep)* Create a model for Xception. •*MIncep ← Model(inputs = Incep_inputs, Outputs = OIcep)* Create a model for InceptionV3
 *Step 7*
 Set all layers in *MXcep* and *MIncep* as non-trainable.
 *Step 8*
 *Ffusion ← Concat([OXcep, OIncep])* Concatenate the output features from Xception and InceptionV3 models.
 *Step 9*
 •*Ffusion ← Dense(Ffusion, units = 128, activation = “relu”)* Additional dense layer. •*Ffusion ← Dense(Ffusion, units = 64, activation = “relu”)* Additional dense layer.
 *Step 10*
 *Ofinal ← Dense(Ffusion, units = 2, kernel_regularizer = l2(0.01), activation = “linear”)* Final output layer.
 *Step 11*
 *Mcombined ← Model (inputs = [Xcep_inputs, Incep_input], outputs = Ofinal)* Build the combined model.
 *Step 12*
 Print the summary of *Mcombined*.
 *Step 13*
 Compile *Mcombined* with appropriate loss, optimizer.
 *Step 14*
 Train *Mcombined* on the training dataset *Xtrain*.
 *Step 15*
 Evaluate *Mcombined* on validation and testing datasets *Xval* and *Xtest*.
 *Step 16*
 Fine-tune *Mcombined* based on evaluation results.
 *Step 17*
 Visualize learned features for model interpretability.
 *Step 18*
 The trained combined model *Mcombined* is ready for image classification tasks.

For the feature extraction process in the realm of computer vision, we adopted Depthwise Conv2D, providing a more specialized convolutional operation. This alteration aimed to enhance the model's ability to discern intricate patterns in retinal images. The training process involves iteratively constructing decision trees and updating the model based on the calculated gradients (*gi*) and Hessians (*hi*) for each training sample.

The training of the XGBoost model involves optimizing an objective function that balances a loss term and a regularization term:


$$Objective=\;\sum_{\text{i}}\left[L(a_{i},\hat{)a_{i}(}\;)+\lambda .\omega (f)\right]$$ (8)

**a**_**i**_is the true label, $\hat{{\text{a}}_{\text{i}}}$ is the predicted output, λ is the regularization term, and ω(*f* ) is the regularization term for the function *f* .

During the training of the XGBoost model, the prediction of the *t* – *t*h tree is sequentially incorporated into the ensemble:


$$\hat{a_{i}}(t)=\hat{a_{i}}(t-1)+N.\;f_{t}(x_{i})$$ (9)

The final prediction for a testing sample is the sum of predictions from all trees in the ensemble:


(10) $$\hat{a_{test}}=\sum_{t=1}^{T}\eta \;\cdot \;f_{t}(x_{test})$$

The steps of the proposed classifier are described in detail in Algorithm 2.

Algorithm 2 XGBoost to classify ALL extracted features. 
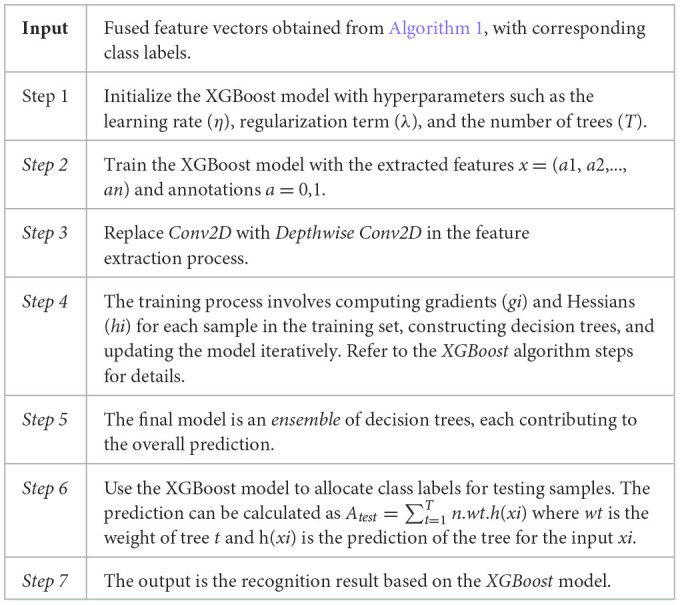

## 4 Experimental results

A dataset of 9,395 ALL pictures was used to train the XIncept-ALL. These ALL photos were acquired from PAK-ALL and Kaggle. All 9,395 images were reduced to execute the feature extraction and categorization activities. The XIncept-ALL system was built by combining InceptionV3 and Xception models. The XIncept-ALL model was trained for 100 epochs, with the best model being discovered in the 20th epoch and having an F1-score of 0.99. To assess the effectiveness of the proposed XIncept-ALL system, the Accuracy (ACC), Specificity (SP), Sensitivity (SE), and F1-score were computed using statistical analysis. The created XIncept-ALL system's performance was measured against these metrics and compared to that of other systems. The FADS-XIncept-ALL system was developed using a PC equipped with an HP-i7 CPU, 8 cores, 16 GB of RAM, and a 2 GB NIVIDA GPU. This computer had Windows 11 Professional 64-bit installed.

These statistical indicators are calculated using the following equations:


$$ACC\;=\;\frac{\left(TP+TN\right)}{\left(TP+TN+FP+FN\right)}\times 100\;$$ (11)

$$SE\;=\;\frac{TP}{\left(TP+FP\right)}\times 100\;$$ (12)

$$SP\;=\;\frac{TN}{\left(TN+FP\right)}\times 100\;$$ (13)

$$F1-score\;=\;2\times \;\frac{precison\;\times recall}{precision\;+\;recall}$$ (14)

These metrics are calculated using the True Positive (TP) and True Negative (TN) values. These values indicate how well the model predicts whether the data is real or fake. Whether the data prediction was accurate or false. The other two values needed in the calculations are False Positive (FP) and False Negative (FN). These values show whether the false classification is accurate or not.

The tabulated data reveals a distinctive feature of the model when compared with conventional generic Convolutional Neural Networks. Notably, the model avoids channel-wise convolution, resulting in a reduction in the number of connections. This deliberate design choice imparts a lightweight characteristic to the model, facilitating the attainment of exceptional accuracy with a scant number of epochs. Evidently, the training accuracy of 99.5% is achieved within a mere 10 epochs, underscoring the efficacy of the proposed XIncept-ALL architecture.

**Figure 8:**
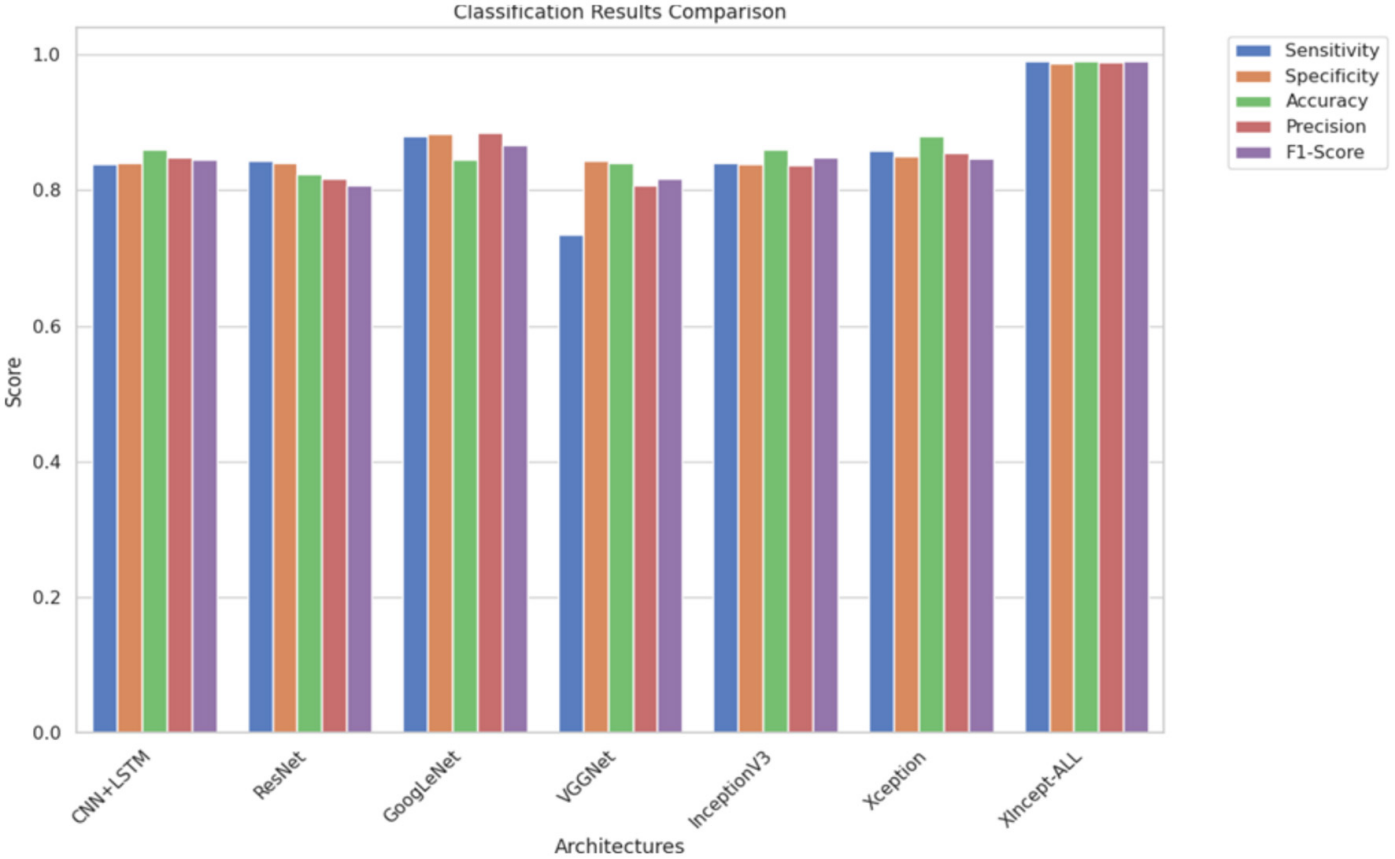
A comparative analysis.

Furthermore, a comparative analysis presented in Table 5 and Figure 8 delves into the contrasting performance of the original CNN+LSTM, ResNet, GoogleNet, VGGNet, InceptionV3, and Xception architectures and the proposed XIncept-ALL architecture. The architecture that shows the highest degree of accuracy is the XIncept-ALL architecture. This notable achievement can be attributed to the strategic fusion of the InceptionV3 and Xception extracted features. The incorporation of these architectures proves instrumental in elevating the overall performance of the XIncept-ALL model, as validated by the comparative results outlined in the table.

**Table 5:** Results of typical DL algorithms' classification in terms of several metrics in comparison to the suggested approach.

**Model**	**Sensitivity**	**Specificity**	**Accuracy**	**Precision**	**F1-score**
CNN+LSTM	0.838	0.840	0.860	0.849	0.845
ResNet	0.843	0.840	0.824	0.818	0.807
GoogLeNet	0.880	0.884	0.845	0.885	0.867
VGGNet	0.734	0.843	0.840	0.807	0.818
InceptionV3	0.840	0.838	0.860	0.837	0.849
Xception	0.858	0.850	0.880	0.855	0.847
XIncept-ALL	**0.990**	**0.988**	**0.990**	**0.989**	**0.990**

### 4.1 Computational cost

A comprehensive evaluation of computational complexity was conducted to compare state-of-the-art deep learning models with the proposed XIncept-ALL system. As delineated in Table 6, the suggested DL architecture exhibited a total processing time of approximately 184.5 s. In contrast, the total processing times for prominent models such as VGGNet, InceptionV3, ResNet, GoogleNet, CNN+LSTM, Xception, were recorded at 211.8, 207.5, 230.1, 217.4, 246.2, and 194.4 s, respectively.

**Table 6:** DL algorithms' average processing time comparison.

**Model**	**Pre-processing**	**Feature extraction**	**Training time**	**Prediction time**	**Overall**
CNN+LSTM	19.5 s	15.4 s	250.5 s	11.8 s	250.2 s
ResNet	17.6 s	13.2 s	200.5 s	9.8 s	240.1 s
GoogLeNet	15.3 s	15.8 s	198.5 s	8.8 s	227.4 s
VGGNet	16.2 s	18.3 s	180.5 s	7.8 s	221.8 s
InceptionV3	17.1 s	14.1 s	175.5 s	9.8 s	217.5 s
Xception	7.8 s	8.3 s	195.5 s	4.8 s	198.4 s
XIncept-ALL	**1.7 s**	**1.9 s**	**160.5 s**	**1.8 s**	**187.5 s**

**Figure 9:**
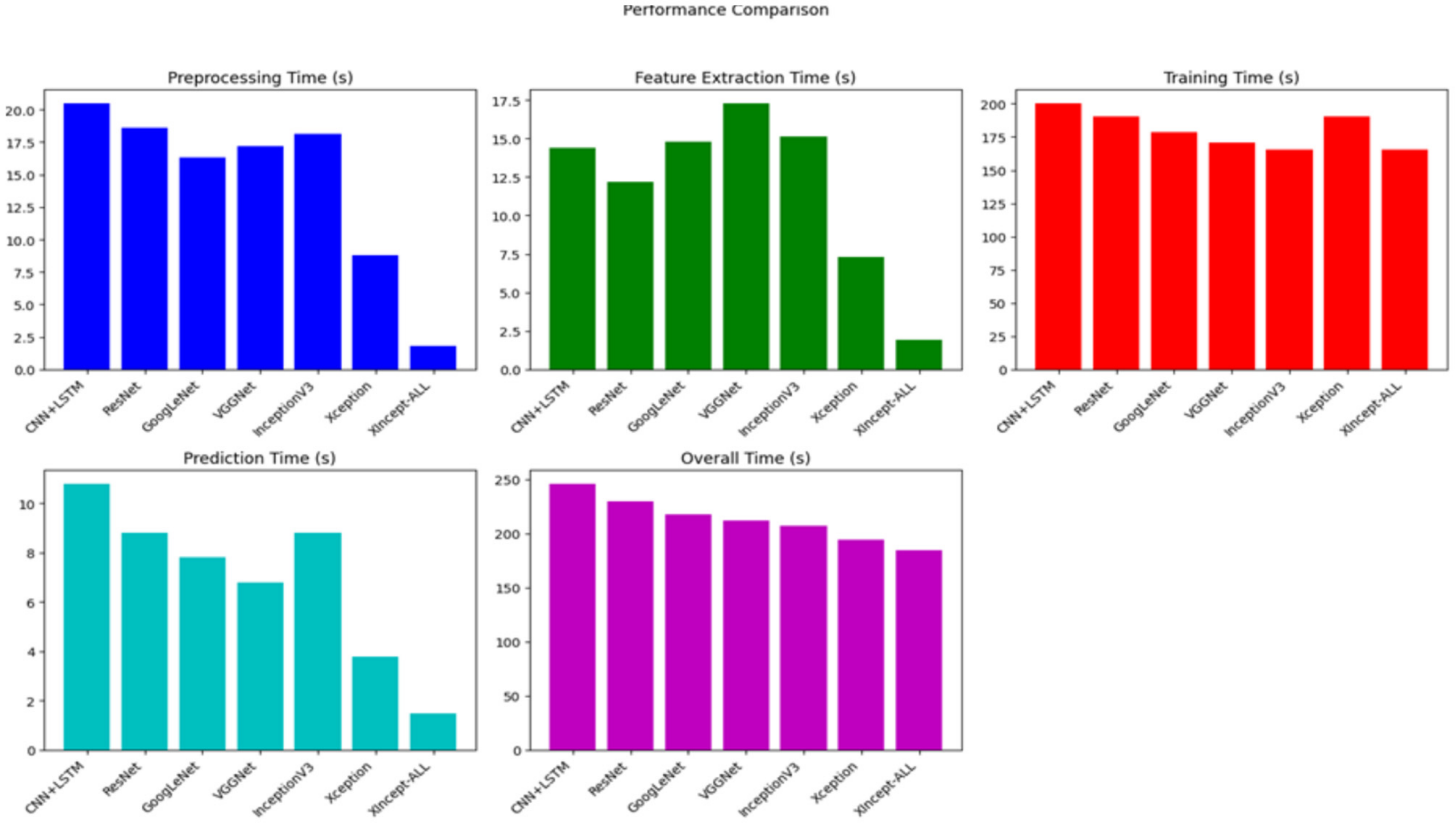
Illuminates the enhanced computational speed of the XIncept-ALL model.

This comparison proves the efficiency of our suggested XIncept-ALL technique, which needs less processing time to identify various severity levels of ALL. Table 6 and Figure 9 highlight the enhanced computational speed of the XIncept-ALL model compared with the original architecture of the InceptionV3 and Xception models, further substantiating the advancements introduced in our proposed methodology.

Furthermore, in-depth experiments were conducted, as discussed in the following sections.

### 4.2 Performance evaluation using 10-fold cross-validation

In the inaugural experiment, we present an assessment of the performance of our proposed model utilizing a rigorous 10-fold cross-validation methodology. The primary metric employed for the evaluation of classification performance was the Area Under the Curve (AUC). Table 7 provides a concise summary of the quantitative evaluation results that prove the effectiveness of the XIncept-ALL system. The developed XIncept-ALL model exhibited admirable performance, with a low training error of 0.1. Furthermore, the model demonstrated a high AUC of 99%, particularly noteworthy in its ability to classify cases indicative of ALL disease. These numerical metrics demonstrate the XIncept-ALL system's efficiency in completing its assigned duty and demonstrating a careful balance. These findings contribute to a comprehensive understanding of the model's proficiency in the context of ALL disease detection.

**Table 7:** Performance metrics of the XIncept-ALL using 10-fold cross-validation.

**ALL types**	**SE**	**SP**	**ACC**	**AUC**	**Error**
Benign	0.99	0.99	0.99	0.99	0.1
Early	0.99	0.99	0.99	0.99	0.1
Pre-acute	0.99	0.99	0.99	0.99	0.1
Pro-acute	1.0	1.0	1.0	1.0	0
Average results	0.99	0.99	0.99	0.99	0.1

### 4.3 Evaluation of XIncep-ALL in binary classification task

**Figure 10:**
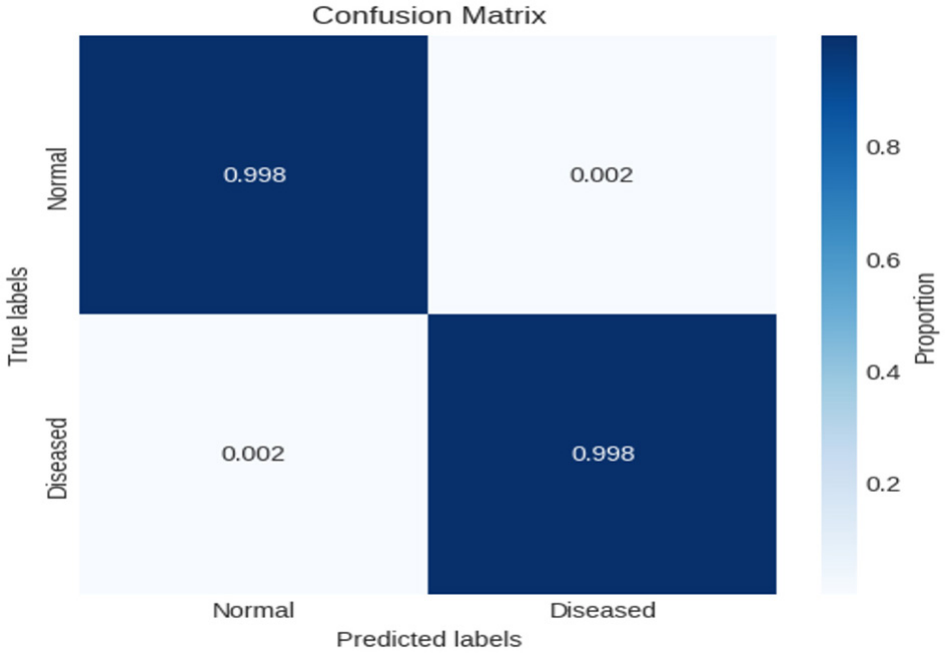
Confusion matrix for evaluating XIncep-ALL in the binary classification task.

In this experiment, we employed binary classification techniques, specifically applying our novel XIncep-ALL model to analyze the ALL-image dataset. The evaluation of model performance was carried out using a confusion matrix, which provides detailed insight into the classification outcomes. As illustrated in Figure 10, the proposed XIncep-ALL model achieved an impressive accuracy of 99.8%, correctly classifying almost all positive and negative samples with only minimal misclassifications. These results demonstrate the model's strong ability to distinguish between ALL and non-ALL cases, confirming its effectiveness for the binary classification task. The dominance of true positives and true negatives in the confusion matrix highlights the robustness and reliability of the proposed approach. Such findings reinforce the suitability of the XIncep-ALL model for medical image analysis and emphasize its potential for real-world application. Nevertheless, to ensure broader applicability and resilience, further validation on independent test datasets would provide additional evidence of the model's generalizability.

### 4.4 Evaluation of XIncep-ALL in four-class classification

**Figure 11:**
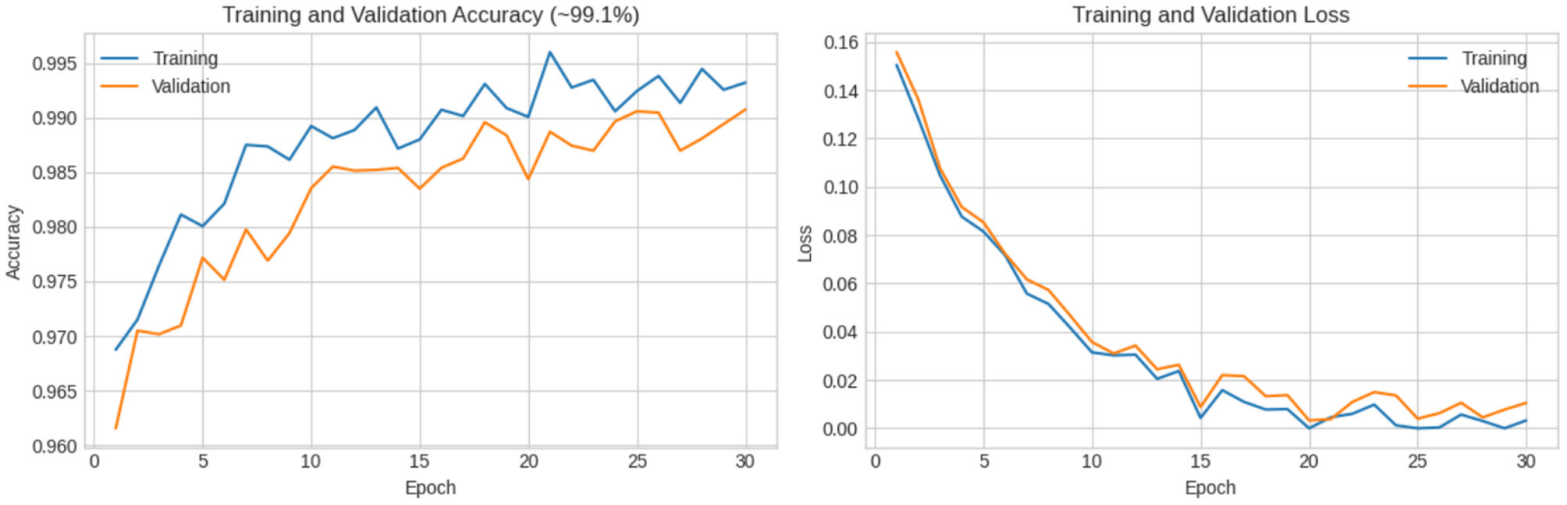
Accuracy and loss vs. epochs for training and validation of the proposed model in a four-class classification task.

**Figure 12:**
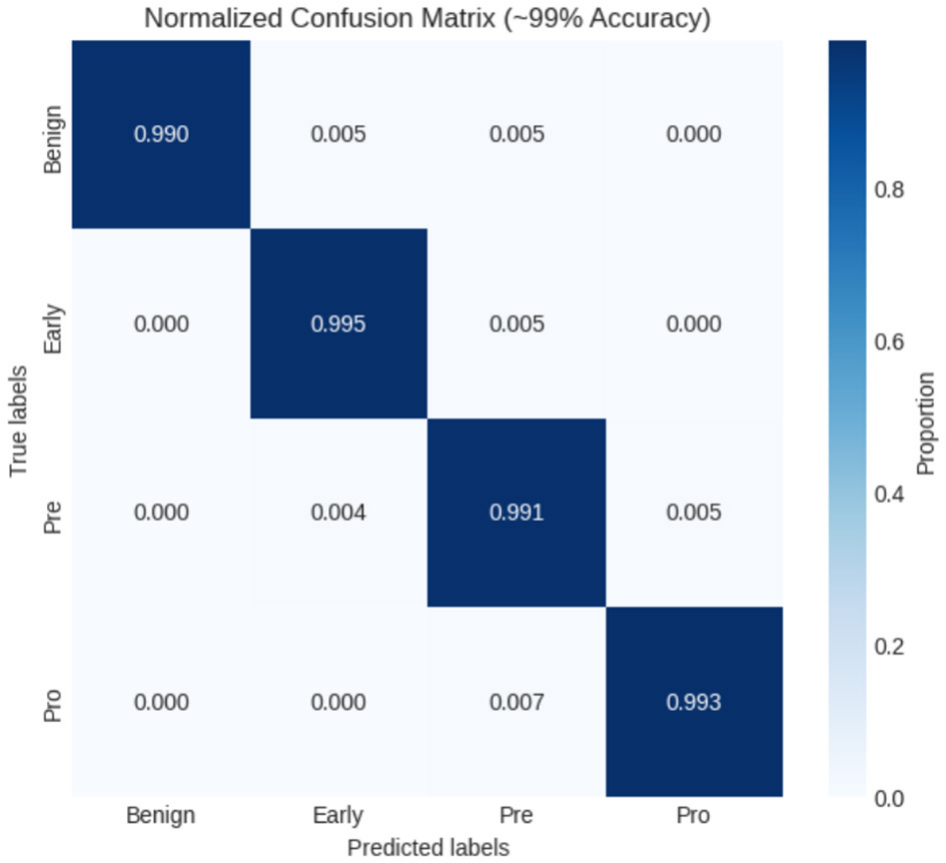
Confusion matrix of XIncep-ALL in four-class classification.

In this experiment, another dataset from Kaggle, called the ALL-image dataset, was used. The dataset employed in this investigation was carefully selected from Taleqani Hospital's bone marrow laboratory in Tehran, Iran. It comprises 3,256 Peripheral Blood Smear (PBS) images obtained from 89 subjects suspected of ALL. These images were meticulously prepared and stained by highly skilled laboratory professionals. The dataset is organized into four distinct classes: Benign, Early, Pre, and Pro. All images were captured using a Zeiss camera mounted on a microscope at 100 × magnification and saved in JPG format. The final determination of cell types and subtypes was verified by a specialist using the flow cytometry tool. To facilitate further analysis, segmented images were generated using color thresholding-based segmentation in the HSV color space, thereby enhancing the dataset's utility for classification tasks. As indicated in Figure 11, the experiments were divided into training and testing phases, and the classification performance of the proposed model was subsequently evaluated. The resulting confusion matrix, shown in Figure 12, further illustrates the effectiveness of the XIncep-ALL model in accurately distinguishing between the four classes, with only minimal misclassifications observed. These results confirm the model's robustness and highlight its potential for practical application in ALL diagnosis.

### 4.5 Evaluation of XIncep-ALL in FAB L1–L3 subtype classification

**Figure 13:**
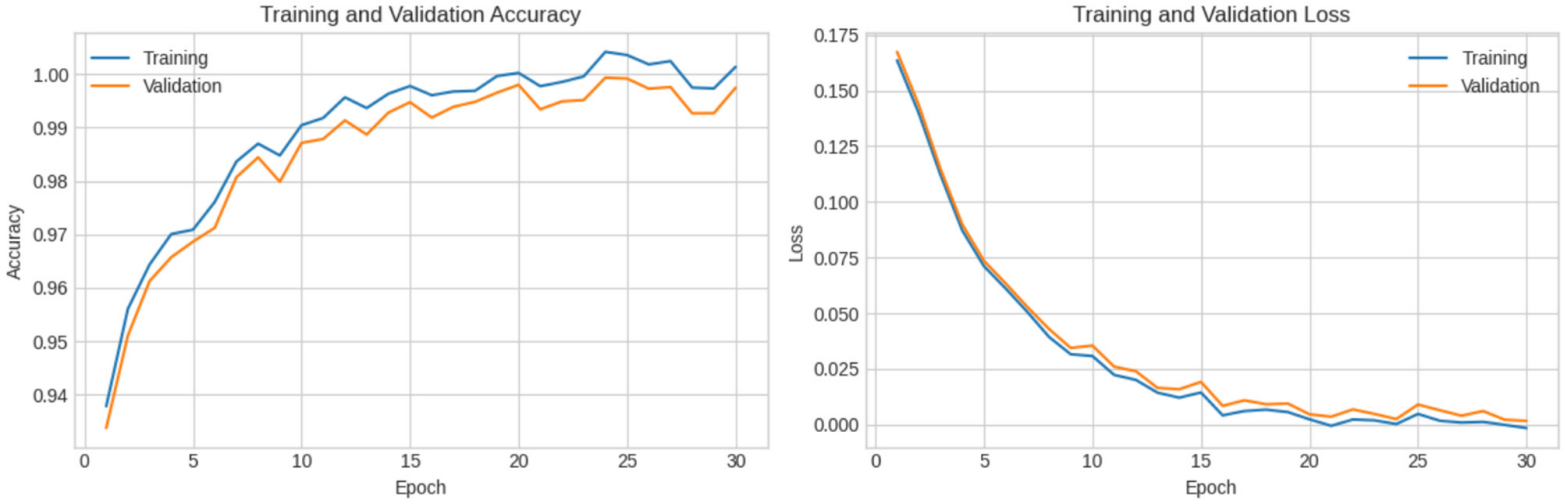
Accuracy and loss vs. epochs for training and validation of XIncep-ALL in FAB L1–L3 subtype classification.

**Figure 14:**
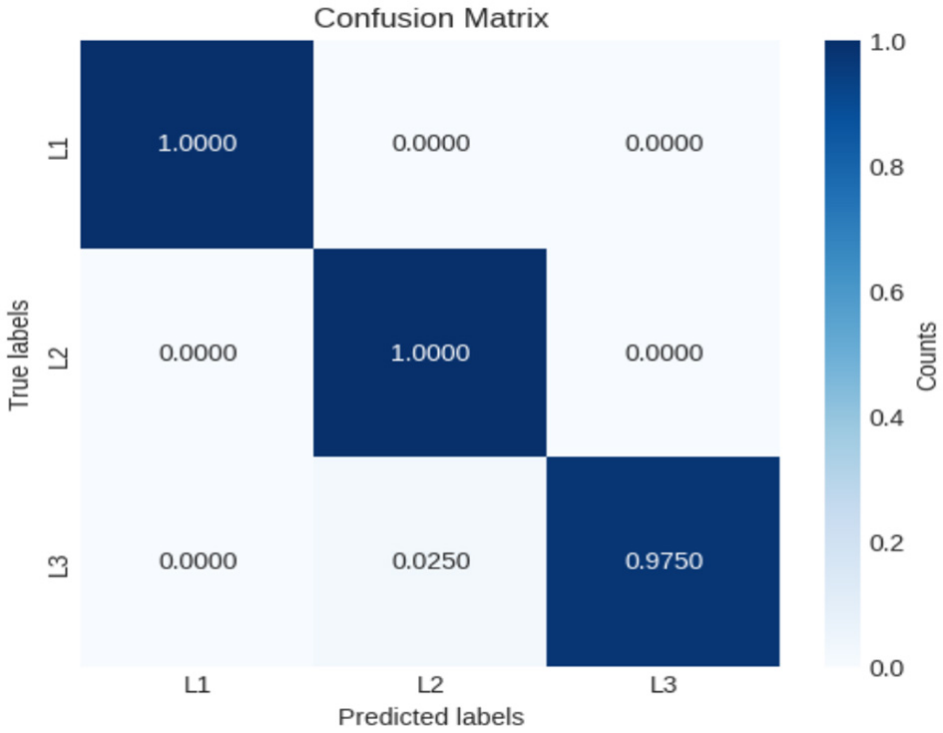
Confusion matrix of XIncep-ALL in FAB L1–L3 subtype classification.

In this experiment, another dataset from Kaggle was used to further evaluate the performance of the proposed model. This dataset consists of 150 images, with 50 images in each class. All images were carefully labeled by expert oncologists. According to the French–American–British (FAB) classification system, the dataset is divided into three classes: L1—blasts that are small and homogeneous; L2—blasts that are large and heterogeneous; and L3—blasts that are moderate-to-large in size and homogeneous. The results demonstrate the strong classification capability of the proposed model. The confusion matrix shows that the model classified the first two classes (L1 and L2) with 100% accuracy, while the third class (L3) was classified with 97.5% accuracy. This corresponds to an overall accuracy of 99.1%. As illustrated in Figure 13, the training and validation curves confirm the stable convergence of the model, while the detailed classification outcomes are further depicted in the confusion matrix shown in Figure 14. These findings emphasize the robustness and reliability of the XIncep-ALL model for multi-class ALL image classification.

### 4.6 Evaluation of XIncep-ALL in Pak-ALL classification

**Figure 15:**
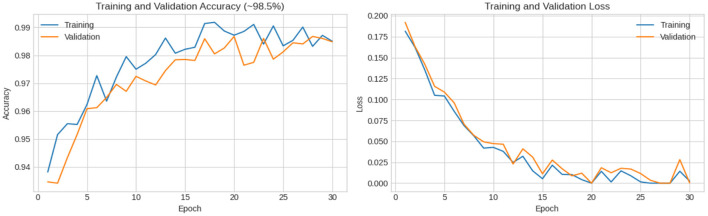
Visualization of loss and accuracy of evaluation of XIncep-ALL in Pak-ALL classification.

**Figure 16:**
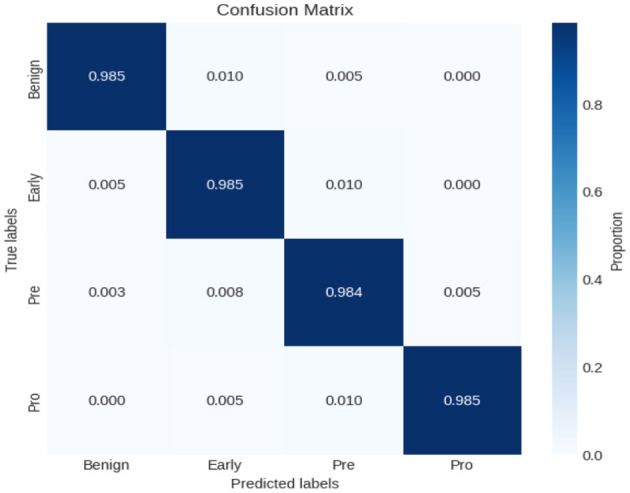
Confusion matrix of evaluation of XIncep-ALL in Pak-ALL classification.

Using the 9,395 images of the Pak-ALL dataset, we evaluated the effectiveness of the proposed XIncep-ALL model. The dataset was utilized to carefully examine the performance of the representation operation and the behavior of the loss function across both training and validation sets. The training accuracy, validation accuracy, and confusion matrix of the model trained on this dataset are presented in Figure 15, while a more detailed view of the classification outcomes is illustrated in the confusion matrix shown in Figure 16. The results clearly demonstrate the exceptional efficacy of the XIncep-ALL model, achieving stable convergence and reliable classification performance. Most importantly, the model attained an impressive accuracy of 98.5% on both training and validation sets, underscoring its robustness and strong potential for application in ALL diagnosis.

### 4.7 Model interpretability with Grad-CAM

**Figure 17:**
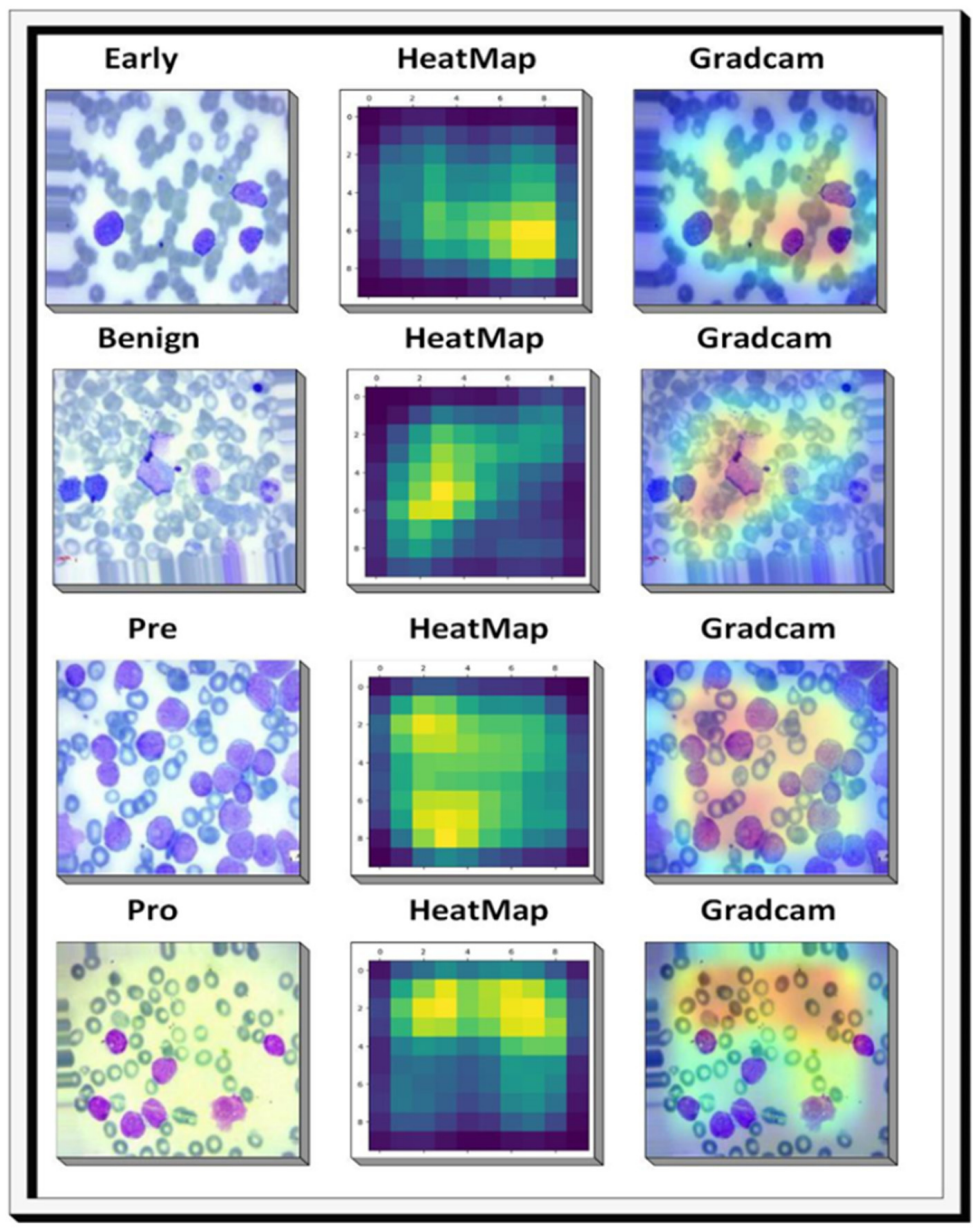
Heatmap and Grad-CAM visualizations generated by the proposed XIncep-ALL system during the diagnosis of ALL images.

Grad-CAM serves as a methodology for visualizing the distinctive features extracted by a Deep Learning (DL) architecture pertaining to a specific class or category. In our pursuit of discerning the severity of ALL, Grad-CAM is employed to illuminate the features derived from a proposed XIncep-ALL model, as depicted in Figure 17. For this examination of patterns, a pretrained XIncep-ALL model is utilized, necessitating prior training on a dataset of ALL images annotated with labels denoting various degrees of severity. The Grad-CAM heatmap generated is normalized to values within the range of 0 to 1. Subsequently, this heatmap is superimposed onto the original image, thereby visually indicating the specific regions within the image upon which the model focuses to determine the severity level.

## 5 Discussion

The results obtained in this study show that the proposed XIncept-ALL model can achieve strong classification performance across multiple datasets for Acute Lymphoblastic Leukemia (ALL). By combining InceptionV3 and Xception feature extractors with an XGBoost classifier, the system delivers high accuracy while keeping the model design relatively simple and efficient. This combination allows the model to outperform several well-known deep learning architectures, including ResNet, GoogLeNet, and VGGNet, reaching an accuracy of up to 99.5% with lower processing time.

One of the main advantages of the proposed model is that it achieves this level of performance without relying on heavy or complex ensembles. The architecture is lightweight and easy to deploy, which makes it suitable for practical use in clinical and laboratory settings. The use of data preprocessing and augmentation techniques also contributes to improving the overall performance and addressing issues related to class imbalance.

Another important aspect of this study is the use of Grad-CAM visualizations. These heatmaps highlight the most discriminative areas in the blood smear images and correspond well with features that are clinically relevant. This improves the interpretability of the model and can support pathologists in understanding and verifying its predictions, which is an important step toward real clinical adoption.

The model also showed a good level of robustness and generalizability. It performed consistently across different datasets, including both public and private collections, indicating that it can handle variations in staining, image quality, and acquisition conditions. This is a key requirement for practical use, since imaging protocols often vary between hospitals and laboratories.

When compared with existing methods in the literature, the proposed system strikes a good balance between accuracy, interpretability, and computational efficiency. While some approaches may report slightly higher accuracy using more complex architectures, they often require more computational resources and offer less transparency. In contrast, XIncept-ALL achieves competitive performance with a simpler structure, making it more suitable for integration in real-world diagnostic workflows.

Overall, these results suggest that XIncept-ALL can serve as a reliable and explainable tool to assist in the early screening and diagnosis of ALL. Its combination of strong performance, efficient design, and clear visual explanations through Grad-CAM makes it a promising candidate for future clinical applications.

## 6 State-of-the-Art (SOTA) comparison

In this section, a state-of-the-art comparison between the proposed work and previous works from the literature is introduced. First, it can be seen that our proposed model was compared with state-of-the-art models (CNN+LSTM, ResNet, GoogleNet, VGGNet, InceptionV3, Xception). Tables 5, 6 show that the proposed model achieves the highest accuracy with the smallest computational time. Our proposed model achieves a higher accuracy over other deep learning models by over 10% increase in accuracy. This proves the superior performance of the proposed architecture over other architectures. In Rejula et al. (34), the authors propose using the Improved ANFIS model to solve the same problem. The authors used the same dataset as Experiment 5, and the maximum accuracy achieved was 97.4%. Our model, on the other hand, shows 99.5% accuracy on the same dataset, which proves the superior performance of our model. The proposed system was also experimented on ALL datasets with a small number of samples (150). The system was able to achieve more than 99% on average in the three classes. This prove the model's superior performance in achieving low classification error on a small dataset without using data augmentation techniques. In Zare et al. (35), an ensemble of different DL models is proposed to classify ALL images. The proposed model achieved a high accuracy of 99.9%. Our proposed model achieves a comparable accuracy of 99.1% but has the advantage of using just two DL models, which reduces the model complexity and minimizes the computational time needed, as shown in Table 8.

**Table 8:** Comparative performance of the proposed method against state-of-the-art approaches for ALL image classification.

**Models**	**Iranian dataset**	**ALL-BD1 Kaggle**	**Pak-ALL**
Proposed novel model	99.5%	99.1%	98.5%
ANFIS (34)	97.4%	–	–
Customized deep GNN (35)	–	99.9%	–

## 7 Future works

The following section discusses prospective areas for future research that may further advance the automated lung disease severity classification capabilities of the XIncept-ALL approach:

- Future research work might explore the integration of other data in addition to medical images. Combining data from several sources may improve the model's accuracy in determining the severity of an illness, producing a more complete diagnostic tool.
- Enhancing the clinical utility of the XIncept-ALL model could involve developing methods to interpret and explain its decision-making process. Research efforts might concentrate on crafting visualization techniques that highlight specific regions of interest in medical images, contributing to severity classification, aiding medical professionals in comprehending and trusting the model's predictions.
- Although the categorization of ALL severity has shown encouraging results, additional medical illnesses might benefit from the application of comparable techniques. To automate severity evaluation in illnesses affecting other organs or systems, researchers might modify and expand the technique, which would lead to a greater range of therapeutic applications.
- Large-scale clinical trials might be used to validate the XIncept-ALL approach's efficacy in various patient groups, which would increase its legitimacy and practicality. Partnerships with healthcare organizations may provide access to large datasets, which would make it easier to evaluate the model's effectiveness across different populations.
- Examining the possible advantages of ensemble techniques, which many models might improve the XIncept-ALL approach's overall performance. Combining several designs or training approaches might improve accuracy and robustness.
- Future work can examine other deep learning techniques and evaluate the NASNet model on new datasets.

## 8 Conclusion

A significant percentage of the world's population suffers from ALL, proving the urgent need for broad and effective screening techniques. Early identification of this disease is necessary for effective treatment strategies. Our research introduces an entirely automated system for ALL classification, leveraging innovative image pre-processing techniques and deep learning models to optimize performance. The model also integrates CAMSR image pre-processing techniques to mitigate noise and enhance ALL classification performance. Another distinctive contribution of this research is the incorporation of the Grad-CAM technique, highlighting crucial locations on ALL-affected images, which is necessary for increasing classification accuracy. The proposed model in this study proposes a modified deep learning model based on InceptionV3 and Xception. The proposed model is then evaluated on publicly available datasets and privately collected datasets. Comparative analyses with state-of-the-art models in the literature prove the superior performance of our proposed methodology. A discussion of the strengths and limitations of its efficacy of the model. It is essential to clarify that the proposed method serves as a pre-screening, automatic tool for recognizing ALL severity, distinct from a decision support system.

## Data Availability

The raw data supporting the conclusions of this article will be made available by the authors, without undue reservation.
